# Epigenetic Regulation of Osteogenic and Chondrogenic Differentiation of Mesenchymal Stem Cells in Culture

**Published:** 2013-05-05

**Authors:** Mohamadreza Baghaban Eslaminejad, Nesa Fani, Maryam Shahhoseini

**Affiliations:** 1 Department of Stem Cells and Developmental Biology at Cell Science Research Center, Royan Institute for Stem Cell Biology and Technology, ACECR, Tehran, Iran; 2 Department of Genetics at Reproductive Biomedicine Research Center, Royan Institute for Reproductive Biomedicine, ACECR, Tehran, Iran

**Keywords:** Mesenchymal Stem Cells, Chondrogenesis, Osteogenesis, Epigenetic

## Abstract

Management of mesenchymal stem cells (MSCs) capabilities to differentiate into osteogenic and chondrogenic lineages would be of utmost importance for their future use in difficult to treat cases of destroyed bone and cartilage. Thus, an understanding of the epigenetic mechanisms as important modulators of stem cell differentiation might be useful. Epigenetic mechanism refers to a process that regulates heritable and long-lasting alterations in gene expression without changing the DNA sequence. Such stable changes would be mediated by several mechanisms including DNA methylation and histone modifications. The involvement of epigenetic mechanisms during MSC bone and cartilage differentiation has been investigated during the past decade. The purpose of this review is to cover outstanding research works that have attempted to ascertain the underlying epigenetic changes of the nuclear genome during *in vitro* differentiation of MSCs into bone and cartilage cell lineages. Understanding such genomic alterations may assist scientists to develop and recognize reagents that are able to efficiently promote this cellular differentiation. Before summarizing the progress on epigenetic regulation of MSC bone and cartilage differentiation, a brief description will be given regarding *in vitro* conditions that favor MSC osteocytic and chondrocytic differentiation and the main mechanisms responsible for epigenetic regulation of differentiation.

## Introduction

Although cells of multi cellular organisms are genetically the same, their functions and structures differ. This diversity is due to the differential expression of genes that originate during development and can be retained through mitosis. Such stable alteration in gene expression is called "epigenetic" since they are heritable in the short term and do not involve the mutation of DNA itself (1).

**Fig 1:**
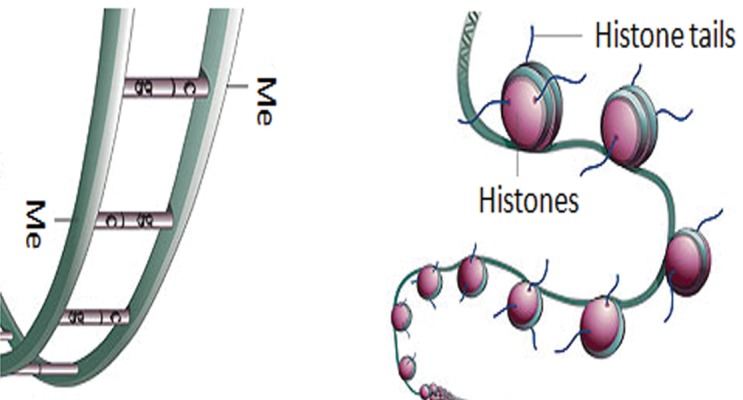
Two main epigenetic modifications of a genome. A. DNA methylation (Me) and B. histone modifications (4).

During the adult life a similar mechanism (longlasting changes in gene expression) occurs during progression from stem cells into differentiated progenies. Differentiation of stem cells into specialized cells requires an up-regulation of genes involved in creation of a specific cell phenotype and suppression of genes responsible for cell stemness (2). Epigenetic regulation of stem cell differentiation refers to the functionally relevant modifications to the genome that do not involve changes in nucleotide sequence. Examples of such changes are DNA methylation and histone modifications (Fig 1) that, in turn, act by modifying the accessibility of genes to transcription factors and other modulators (3, 4).

Mesenchymal stem cells (MSCs) are adult stem cells that possess two major properties, self-renewal ability and the potential for multilineage differentiation. Although MSC have been originally isolated from bone marrow, (5, 16) further investigation has shown that multiple tissues contain MSC-like populations (7-16). Reportedly, the most important characteristics of MSCs are their potential for differentiation into bone and cartilage cell lineages (5, 6). This capacity has generated tremendous excitement for the regeneration of damaged bone and cartilage tissues that are either incurable or difficult to cure due to insufficiency or failure of current therapies (17-20). Generally, there two strategies for the application of MSCs in regenerative medicine. One strategy uses cells in an undifferentiated state, which allows them to undergo differentiation atthe defective site. The disadvantage of this strategy is the unwanted differentiation of cells at the repair site. For instance, if MSCs are to be used for the regeneration of cartilage tissue, bone cells may be produced by unwanted cell differentiation. An alternative approach is to fully differentiate MSCs into the desired cells prior to their transplantation (21, 22). With this strategy, the *in vitro* differentiation of MSCs into bone and cartilage cell lineages seems to be an inevitable step prior to their application in the cell-based treatment of tissue defects. Therefore, the differentiation process of MSCs must be thoroughly understood, particularly in terms of its regulatory mechanisms.

From the discovery of MSCs until now, numerous attempts have been made to understand their differentiation process. Particularly, research has focused on differentiation into bone and cartilage cell lineages the *in vitro* conditions favoring MSC bone and cartilage differentiation. Furthermore, gene expression profile during progression from stem cell into bone and cartilage cells are mostly revealed (reviewed below). Another issue related to MSC differentiation is the epigenetic regulation underlying their osteocytic and chondrocytic differentiation of which investigations have recently begun. The purpose of this paper is to briefly review the main epigenetic mechanisms including DNA methylation and histone modifications, to summarize all studies that have attempted to determine the underlying epigenetic changes of the nuclear genome during MSC bone and cartilage differentiation, and finally to highlight the importance of epigenetic studies in bone and cartilage engineering and regenerative medicine. First, a brief description will be given regarding *in vitro* conditions necessary for osteocytic and chondrocytic differentiation of MSCs and the main transcription factors that promote tissue-specific gene expression during differentiation.

### *In vitro* bone differentiation

*in vitro* bone differentiation of MSCs is a complex process requiring multiple soluble inducers. To establish an osteogenic culture, a confluent monolayer culture of MSCs must be prepared and provided with osteogenic medium, which typically consists of a basal medium such as Dulbecco’s modified eagle medium (DMEM) supplemented with osteogenic inducers. The most-frequently used osteogenic supplement is composed of dexamethasone (10 nM), ascorbic acid (50µg/ml) and β-glycerol phosphate (10 mM). Dexamethasone is the essential component; its continual supplementation is required for human MSC ostegenic differentiation (23). Ascorbic acid, another osteogenic component, is not essential for MSC bone differentiation but its addition enhances production of collagen-rich extracellular matrix (ECM) (24). βglycerol phosphate in the osteogenic medium provides favorable conditions for culture mineralization (25, 26).

In addition to the above mentioned frequently used reagents, other factors that impact MSC differentiation into a bone cell lineage include 1, 25-dihydroxyvitamin D3 (27) and estrogen (28). According to some studies parathyroid hormone (PTH) exhibits an osteogenic effect on MSCs if the culture is exposed intermittently to PTH (29, 30). Local factors including prostagland in,transforming growth factor-beta (TGF-β), fibroblast growth factor-2 (FGF-2) and bone morphogenetic proteins (BMPs), particularly BMP6, have been reported to promote *in vitro* MSC osteogenesis (31-33). Other factors which have osteogenic effects include lithium chloride (LiCl) and 6-bromoindirubin-3΄-oxim (BIO) (33). Additionally, melatonin, a hormone secreted by the pineal gland exhibits osteogenic effects on MSC culture (34). The osteogenic factors thus far mentioned are more effective when used synergistically. For example, it has been shown that addition of BMP2 into a rat MSC culture enhanced the osteogenic potency of FGF-2. Dexamethasone and vitamin D3 as well as BMP2 and retinoic acid have been shown to exhibit a synergistic effect on MSC osteogenic culture (35-37).

Osteogenic supplements of the MSC monolayer culture eventually lead to expression of specific osteoblastic transcription factors. Core binding factor alpha 1 (Cbfa1), which is also called Runx2, is one of the most studied transcription factors expressed in MSCs upon their commitment toward an osteogenic differentiation (38, 39). Upon expression, Runx2 must be activated through posttranslational modifications or protein-protein interactions (40). Other transcription factors may collaborate with Runx2 to promote osteogenic differentiation. It has been found that TAZ, a transcriptional co-activator, co-activates Runx2-dependent gene transcription in murine MSCs (41). Runx2 activates the expression of bone-related genes, including osteocalcin, collagen I, osteopontin, bone sialo protein and the parathormon receptor (PTHR) (39).

Osterix is another transcription factor whose involvement has been discovered in MSC bone differentiation. This discovery was particularly notable in murine MSCs transduced with the osterix gene (42).

### *In vitro* cartilage differentiation

The induction of chondrogenesis in MSCs depends on the coordinated activities of two fundamental parameters: cell density and growth factors (43-46). The TGF-β super family of proteins and their members, such as BMPs are established regulatory factors in chondrogenesis. TGF-β promotes proteoglycan deposition, so that in its absence the ECM of differentiated cells contains modest amounts of proteoglycan (47). TGF-β_1_ is a standard media additive used in cultures to induce chondrogenesis. TGF-β3 has been shown to induce a more rapid, representative expression of a chondrogenic culture (48, 49). In the cell laboratory, cartilage differentiation of MSCs can be performed in a pellet culture system. Approximately 2 × 105 cells (passages 2-3) must be condensed in to a pellet by centrifugation at 300 g for 4 minutes, followed by incubation in an atmosphere of 37˚C and 5% CO_2_ in a 0.5 ml chondrogenic medium. The chondrogenic medium should be composed of 10 ng/ml TGF-β3, 500 ng/ml BMP-6, 100 nM dexamethasone, 50 µg/ml ascorbic 2-phosphate, 50 µg/ml ITS and 1.25 mg/ml bovine serum albumin. Recently, we have shown that addition of Lithium Chloride and a small molecule refereed to as SB216763 can enhance glycoseaminoglycal deposition in the human marrow-derived MSC chondrogenic culture (50).

Sox9 is the main transcription factor essential for chondrocyte differentiation of MSCs. In the chondrogenic culture of MSCs. Expression of Sox9 is followed by chondrocyte-specific gene expression that includes collagen I and aggrecan. Genetic mutations in *Sox9* leads to congenital dwarfism syndrome (51).

### Epigenetic mechanisms

#### DNA methylation

Currently, one of the epigenetic changes mostly studied in mammals is DNA methylation, which primarily involves the establishment of parental- specific imprinting during gametogenesis (52). This process includes covalent binding of a methyl group from a methyl donor, mainly S-adenosylmethionine, to carbon 5 of the cytosine that often is located in the CpG sites. This enzymatic reaction is produced by a family of enzymes called DNA methyltransferases (Dnmts) (53). There are several types of Dnmts, including de novo Dnmt3a and Dnmt3b,which are highly expressed in the developing mouse embryo and promote global de novo methylation after implantation (54). Dnmt1 is a methyltransferase that maintains the existing methylation patterns upon cell division (52). Genomic regions that contain a high number of methylated cytosine are usually transcriptionally inactive. The absence of DNA methylation is a prerequisite for transcriptionally active genes (55, 56).

#### Histone modifications

Histones, the major structural proteins of chromosomes, are small proteins that contain numerous positively-charged amino acids such as lysine and arginine. These positively charged amino acids enable histones to tightly bind with the phosphatesugar backbone of double stranded DNA. These proteins have a tail comprised of a long aminoacid chain in their N-terminal domain that plays an important role in regulation of chromatin structure. The histone tail domains are considered as master control switches that define the structural and functional characteristics of chromatin at many levels. These structures modulate DNA accessibility within the nucleosome and are essential for stable folding of oligonucleosome arrays into condensed chromatin fibers (57). Histone tails may have varying fates including acetylation, methylation, phosphorylation, polyadenylation, ribosylation, ubiquitination and glycosylation. Combinations of these modifications determine the overall interaction of histones with the DNA molecule, leading to activation and/or inhibition of transcription (58). Of these, acetylation and methylation are the mostepigenetic mechanisms studied in transcriptional regulation.

**Fig 2:**
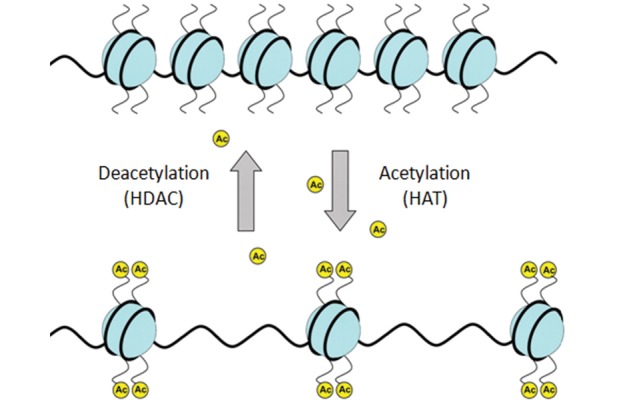
Histone acetylation and deactylation. Histone acetyltransferase (HATs) adds acetyl groups (Ac) onto histone tails,which results in a nucleosome openingthus allowing for transcription factors to access DNA and initiate gene transcription.Histone deacetylases (HDACs) remove the Ac from the histone tails, leading to a closed chromatin structure (61).

Acetylation is one of the studied histone modifications that occurs primarily at the lysine of histones 3 and 4, and is basically catalyzed by acetyltransferase enzymes such as HBO1, TIP60, MORF/Moz and MOF. The consequence of this modification is the loss of the positive charge of the lysine residue which affects the histone’s binding to the DNA molecule,and is defined as nucleosome opening (Fig 2). Acetylation levels of histone tails dependent on balance between the two enzymatic activities of acetyltransferase and deacetylase (58). There are four classes of histone deacetylase (HDAC). Class I includes HDAC 1, 2, 3, and 4. Class II is comprised of HDAC5, 6, 7, 9, and 10. Class III includes Sirtuin 1-7 and class IV includes HDAC11. Among these, the HDAC of classes I, II and IV have the same sequences and structures. Sirtuin, however, has a different structure and a different catalytic mechanism. Sirtuin proteins comprise a unique class of NAD ± dependent protein deacetylases (59).

Acetylation of the histone tails leads to neutralization of the partial electric charge of lysine which in turn results in opening of the chromatin structure. *in vitro* observation of this event is not a simple task, but biophysical analysis has shown that intranuclosomal linkages are important for chromatin stabilization. According to research, acetylation of lysine 9 on histone 3 has a dominant negative effect on the formation of 30 nanometer chromatin fibers and higher-order structures (60).

Acetylation of the histone tails leads to neutralization of the partial electric charge of lysine which in turn results in opening of the chromatin structure. *in vitro* observation of this event is not a simple task, but biophysical analysis has shown that intranuclosomal linkages are important for chromatin stabilization. According to research, acetylation of lysine 9 on histone 3 has a dominant negative effect on the formation of 30 nanometer chromatin fibers and higher-order structures (60).

Lysine can be mono-, di-or tri-methylated but argentine can be only mono-methylated.The level of histone methylation is controlled by the dual enzymatic activities of methyl transferase and demethylase (64). Basically, there are two classes of proteins that include thepolycomb group and tritorax group complexes which act as methyl transferase elements during development. These histone methylating enzymes encode methylation of lysine 27 and lysine 4 of histone 3, respectively. It has been shown that a precise balance between these two enzymatic activities modulates epigenetic regulation of cellular differentiation processes (58).

#### Epigenetics of bone differentiation

Over the past decade, several researchers have investigated epigenetic control of MSC bone differentiation. In this context and according to numerous research DNA methylation is dynamically involved in the process of bone differentiation of MSCs. For example, Villagra et al. have observed a significant hypermethylation at the *osteocalcin* gene locus in undifferentiated cells, which was associated with the condensed chromatin structure. Their subsequent examination has revealed that during *in vitro* osteoblast differentiation, CpG methylation of the *osteocalcin* promoter significantly decreased as the *osteocalcin* geneu pregulated (65).

Arnsdorf et al. have designed a novel protocol to promote MSC osteogenic differentiation by the application of a mechanical stimulus. Following successful differentiation they attempted to determine the possible underlying mechanism of MSC osteogenesis. According to their results, the increase observed in bone-specific gene expression was under the control of epigenetic regulation of several osteogenic candidate genes. Mechanical stimulation of MSCs reduced the DNA methylation state of the genes, which lead to their increased expression (66).

Involvement of DNA methylation in osteogenic differentiation of MSCs has also been reported by Dansranjavin et al. (67). They demonstrated that differentiation of MSCs into osteoblast and adipocyte cells was accompanied by reduced expression of the stemness genes such as Brachyury and *LIN_2_8*, which basically occurred via hypermethylation of their promoter regions (67).

Hsiao et al. have observed epigenetic regulation of the thyroid hormone receptor interactor 10 (Trip 10) during osteogenic induction of human bone marrow-derived MSCs. To determine whether DNA methylation-induced gene silencing was involved in this process, they applied an *in vitro* method that specifically methylated the Trip 10 promoter. The transfection of exogenous methylated Trip 10 promoter DNA into MSCs resulted in progressive accumulation of methyl-cytosines at the endogenous Trip 10 promoter, reduced Trip 10 expression, and accelerated MSC-to neuron and MSC-to-osteocyte differentiation (68).

Histone acetylation is another epigenetic mechanism reported to be involved in osteogenesis. Shen et al. have investigated the chromatin-mediated mechanisms by which the bone-specific *osteocalcin* gene is transcriptionally activated during cessation of cell growth in ROS 17/2.8 osteosarcoma cells, as well as during normal osteoblast differentiation (70). They assayed acetylation of histones H3 and H4 at the *osteocalcin gene* promoter during and after cell proliferation by using the chromatin immunoprecipitation (ChIP) technique. These researchers observed that both the promoter and coding region of the osteocalcin gene contained high levels of acetylated H3 and H4 histones during the proliferative period of osteoblast differentiation. According to their findings active expression of the osteocalcin gene in mature osteoblast and confluent ROS 17/2.8 cells is functionally linked to preferential acetylation of core histones (70). In contrast, Tan et al. have used microarrays to investigate the roles of histone modifications (H3K9Ac and H3K9Me2) upon the induction of human MSC osteogenic differentiation. In their research, enrichment of H3K9Acglobally decreased at the gene promoters whereas the number of promoters enriched with H3K9Me2 increased upon bone differentiation (71). We have attributed the discrepancies in these two reports to the difference in the cells (cell line or MSCs) and the method (ChIP or microarray) used in each experimental design.

Others, however in order to study the reverse role of histone deacetylation in osteogenes is preferred to measure the acetylation/deacetylation process. Lee et al. examined the expression level of HDAC and degree of histone acetylation at the promoter regions of osteoblast genes. They have noted that down-regulation of HDAC1 is an important process for osteogenesis (72).

Histone methylation has also been reported as an epigenetic mechanism underlying MSC osteogenic differentiation. In this context Hassan et al .have found that *HOXA10* (a gene necessary for embryonic patterning of skeletal elements) contributes to osteogenic lineage determination through activation of Runx2, alkaline phosphatase, osteocalcin and bone sialoprotein (73). Their further investigations have revealed that these effects are mediated through total chromatin hyperacetylation and H3K4 hypermethylation of the genes. In this context, Fan et al. have found that the BCL-6 corepressor (BCOR) mutation increases histone H3K4 and H3K36 methylation in MSCs. This, in turn, reactivates transcription of the *osteo-dentinogenic* gene in MSCs. In their study MSCs were isolated from a patient with oculo-facio-cardio-dental (OFCD) syndrome which is the result of a mutation in the *BCOR* gene. This syndrome is characterized by canine teeth with extremely long roots, congenital cataracts, craniofacial defects, and congenital heart disease (74).

Involvement of histone methylation in MSC bone differentiation is also supported by the work of Wei et al. These authors have found that the activation of cyclin-dependent kinase 1 (CDK1) promotes MSC bone differentiation through phosphorylization of theenhancer of the zeste homologue 2 (EZH2) which is the catalaytic subunit of the polycomb repressive complex 2 (PRC2) that catalizes trimethylation of histone H3 on Lys 27 (H3K27) at Thr 487 (75).

Thus, according to the above-mentioned studies, several epigenetic regulations that include DNA methylation, histone acetylation and methylation might involve MSC osteogenic differentiation. It is not clear whether all three mechanisms are simultaneously involved during MSC bone differentiation or if only one mechanism promotes differentiation dependent on the culture conditions. This issue needs additional investigation.

#### Epigenetics of cartilage differentiation

Few studies have been conducted with regards to epigenetic regulation of gene expression during MSC cartilage differentiation. The work by Ezura et al. (76) isnotable. These authors have investigated the CpG methylation status in human synovium-derived MSCs during *in vitro* chondrogenesis and found that DNA methylation levels of CpG-rich promoters of chondrocyte-specific genes were mostly maintained at low levels (76).

There are many investigations in which the epigenetic mechanism involved in cartilage differentiation has been investigated by the use of chondrocyte or relevant cell lines. Histone acetylation is among theepigenetic mechanisms that have been reported to be involved in cartilage-specific gene expression. In this context the role of p300, an enzyme possessing a histone acetyltransferase (HAT) activity, was observed in several studies. Using the chondrosarcoma cell line SW1353, Tsuda et al. have shown that Sox9 associates with CREB-binding protein (CBP)/p300 via its carboxyl termini activation domain and functions as an activator for cartilage tissue-specific gene expression during chondrocyte differentiation (77). Later, Furumatsu et al. have investigated the molecular mechanism of synergy between Sox9 and p300 in chromatin mediated transcription on chromatinized templates *in vitro*. Their results revealed that p300 potentiated Sox9-dependent transcription through hyperacetylation of histones. P300/ CBP acts as a coactivator to cartilage homeoprotein- 1 (Cart1) through acetylation of the conserved lysine residue adjacent to the homeodomain (78). This point has been mentioned by Iioka et al. who have conducted a study using an *in vitro* acetylation assaythat investigated the functional involvement of p300/CBP during chondrogenesis. Cart1 is expressed selectively in chondrocyte lineage during embryonic development (79).

Histone deacetylation by HDAC1 has been reported to have a critical inhibitory role in cartilage noncollagenous matrix deposition during cartilage differentiation. Cartilage oligomeric matrix protein (COMP) is a noncollagenous matrix protein in cartilage. In a study using Sox-9-null mice, Liu et al. in 2007 have shown that the COMP gene was inhibited by a transcription repressor,the negative regulatory element (NRE)-binding protein by recruiting HDAC1 to the *COMP* promoter (80). In another study by the same authors on rat chondrosarcoma cells and BMP-2-treated C3H10T1/2 progenitor cells, it was observed that the leukemia/ lymphoma-related factor, a POZ domain-containing transcriptional repressor, interacted with HDAC1 and inhibited COMP gene expression and chondrogenesis (81).

Using HDAC4-null mice, Vega et al. have found that HDAC4 regulates chondrocyte hypertrophy and endochondral bone formation by inhibiting the activity of Runx2 which is a transcription factor necessary for chondrocyte hypertrophy. It has been shown that HDAC4-null mice display premature ossification of developing bone; and conversely, over expression of HDAC4 in proliferating chondrocytes *in vivo* inhibits chondrocyte hypertrophy and differentiation (82).

In contrast to deacetylation, histone acetylation favors cartilage differentiation which has been shown in both *in vivo* and *in vitro* studies conducted by Hattori et al. These authors have conducted a study to determine Sox9-regulated gene transcription during chondrogenesis. In this study, they have found a specific interaction between Sox9 and Tat interactive protein-60 (Tip60) which leads to enhanced acetylation of Sox9, mainly through the K61, 253, and 398 residues and subsequent enhancement of its transcriptional activity (83).

In some studies, results have shown that activity of HDAC in cartilage differentiation is mediated through the Wnt signaling pathway. In this context Huh et al. have investigated the role of HDAC in the expression of type II collagen that is a marker of differentiated chondrocytes. They have found that HDAC activity in a primary culture of articular cartilage decreased during dedifferentiation that had been induced by serial monolayer culture; the activity was recovered during 3-D culture. It was also observed that HDAC inhibition promoted the expression of Wnt-5a which is known to inhibit type II collagen expression. Conversely, knockdown of Wnt-5a blocked the ability of HDAC inhibitors to suppress collagen II expression. They have concluded that HDAC promotes collagen II expression by suppressing the transcription of Wnt-5a (84).

In conjunction and according to a study on MSCs, during chondrogenic differentiation DNA methylation levels of CpG-rich promoters of the chondrocyte- specific genes are mostly maintained at low levels. Conflicting reports exist for non-MSCs, however numerous studies have reported an association between histonehyperacetylation and chondrogenic differentiation, (78, 79) or the inhibition of cartilage differentiation by histone deacetylation (80-83). Some researchersbelieve that cartilage differentiation is associated with histone deacetylation (84).For further clarification of the subject, additional research must be performed using MSCs.

#### Application of epigenetics in bone and cartilage engineering and regeneration

 The knowledge obtained by epigenetic studies on MSC osteocytic/chondrocytic differentiation could be applied to bone and cartilage engineering as well as regenerative medicine. As mentioned earlier, epigenetic modification is the process of adding and removing chemical tags,i.e. acetyl or methyl groups, on DNA or its surrounding histones which results in activation or suppression of the genes involved in stem cell differentiation. On the other hand the key process in MSC-based bone and cartilage engineering is to efficiently direct the cells into differentiated phenotypes within an appropriated 3-D scaffold. After identification of epigenetic tags underlying MSC bone and cartilage differentiation, the next step would be to locate suitable chemicals or pharmaceuticals that are able to promote those epigenetic modifications. By using these reagents appropriate bone and cartilage constructs could be developed. Such constructs could be used for transplantation into large bone and cartilage defects which are considered to be problematic in the field of orthopedics.

## Conclusion

MSCs are considered as promising cell candidates for future treatment of difficult bone and cartilage defects. Some scientists believe that transplantation of MSCs at the differentiated state would be more advantageous than transplantation at the undifferentiated state. Thus, investigations of MSC osteogenic and chondrogenic differentiation are of utmost importance. One objective of this research would be to define the precise condition under which MSC differentiation can occur in a controlled, predictable manner. Understanding epigenetic control of cell differentiation will certainly enable scientists to achieve this goal. In this context, promising progress has been made after approximately a decade of research. It has been revealed that DNA methylation, as well as histone acetylation and methylation are involved in MSC bone differentiation.

In the context of cartilage differentiation of MSCs, to the best of our knowledge, there are few studies that have been performed. Most have been conducted using chondrocytic cells or related cell lines. According to these, predominantly DNA methylation and histone acetylation are involved in the control of cartilage differentiation. Understanding the epigenetic mechanism that regulates cell differentiation may result in the development of an appropriate reagent or enzyme that could promote the necessary epigenetic changes of the genome required for efficient differentiation of MSCs. This, in turn, would be considered the preferential cellular material with which to regenerate large defects in bones and cartilages.
